# The influence of the Big Five inventory on quality of life in people with Parkinson’s disease aged 50 and above: A Longitudinal Analysis from the Survey of Health, Aging and Retirement in Europe (SHARE)

**DOI:** 10.1371/journal.pone.0322089

**Published:** 2025-05-30

**Authors:** Sarah Mendorf, Konstantin G. Heimrich, Hannah M. Mühlhammer, Aline Schönenberg, Tino Prell

**Affiliations:** 1 Department of Neurology, University Hospital Jena, Jena, Germany; 2 Department of Geriatrics, University Hospital Jena, Jena, Germany; 3 Department of Geriatrics, University Hospital Halle, Halle, Germany; UCL: University College London, UNITED KINGDOM OF GREAT BRITAIN AND NORTHERN IRELAND

## Abstract

**Background:**

Parkinson’s disease (PD) significantly reduces quality of life (QoL), particularly due to its complex interplay of motor and nonmotor symptoms. While personality traits influence QoL in chronic diseases, their longitudinal effects in people with PD (PwPD) remain underexplored. This study evaluates the longitudinal predictive influence of neuroticism, conscientiousness, and openness on QoL in PwPD over two waves of the Survey of Health, Aging, and Retirement in Europe (SHARE).

**Methods:**

This study utilized longitudinal data from 100 PwPD participants in waves 7 and 8 of the Survey of Health, Aging, and Retirement in Europe (SHARE). QoL was assessed using the CASP-12 scale, while personality traits were measured with the Big Five Inventory (BFI-10). Linear regressions and generalized estimating equations (GEE) were used to examine cross-sectional and longitudinal associations between personality traits and QoL, controlling for sociodemographic, psychosocial, and health-related variables.

**Results:**

Neuroticism was consistently associated with lower QoL across all analyses. Cross-sectional results showed neuroticism as the strongest predictor of QoL decline in wave 7 (beta = -0.33, p < 0.001), and longitudinal GEE analyses confirmed its predictive effect (beta = -0.03, p = 0.007). Conscientiousness and openness showed limited and inconsistent associations with QoL. Beyond personality traits, depressive symptoms and mobility limitations were found to substantially impact QoL, influencing the effects of neuroticism.

**Conclusions:**

Neuroticism plays a pivotal role in predicting QoL decline in PwPD, highlighting its utility as a target for psychological interventions aimed at emotional regulation and resilience building. While depressive symptoms and mobility limitations also contribute, integrating personality assessments into care strategies may improve outcomes. These findings advocate for a multidimensional approach to managing PD that addresses both clinical and psychological factors.

## Introduction

Parkinson’s disease (PD) is a neurodegenerative disorder that affects millions of people worldwide. It causes a wide range of motor and nonmotor symptoms [1]. While the physical manifestations of PD have been investigated in detail, recent research has gone beyond physical health to focus on the overall quality of life (QoL) of people with PD (PwPD).

The concept of QoL refers to how individuals perceive their physical, emotional, and social well-being [2,3]. While health-related QoL encompasses solely those factors that are part of an individual’s health [4], overall QoL is a comprehensive concept that encompasses all factors affecting an individual’s well-being. This also includes mental health and social participation. Of note, due to the complexity of PD and the high prevalence of both motor and nonmotor symptoms such as depressive mood, cognitive deficits and social withdrawal, it is beneficial to assess general QoL in PwPD in order to evaluate their overall well-being instead of merely assessing health-related QoL. This is because some studies suggest that in PD, nonmotor symptoms may affect QoL more strongly than motor symptoms [5,6]. Thus, studies have shown that PwPD generally have a lower QoL compared to the general population [7], despite some studies reporting no significant differences in the physical health domain of QoL [8,9].

Several factors have been reported to influence QoL in PwPD, such as age, sex, education, limitations in activities of daily living (ADL) and instrumental activities of daily living (IADL), depression, mobility limitations, cognition, marital status, general health and, personality traits [7,10–16]. Such within-person characteristics like attitudes towards health, self-efficacy and personality traits play a key role for QoL, as they modify how health and life circumstances are perceived and dealt with [17,18].

**Health-related variables and QoL in PD**

- Self-Rated Health (SRH): In PwPD, SRH is influenced by symptom perception and disease progression, often outweighing objective health measures [19]. Interventions like mindfulness may mitigate the impact of neuroticism on QoL [20].
- Depressive Symptoms: Depression significantly worsens QoL in PwPD [16]. Effective psychological and pharmacological management is crucial [21].
- Functional Limitations: ADL and IADL limitations, worsened by non-motor symptoms, are key factors in reduced QoL, emphasizing the need for targeted interventions [5].

**Demographic variables and QoL**

- Age and Sex: Older age and greater disease severity lower QoL [7], with women often experiencing worse QoL due to higher non-motor symptom burdens [10].
- Education and Marital Status: Higher education and stable marital relationships improve QoL by enhancing access to resources and social support [22]. Socioeconomic factors play a vital role in managing chronic diseases like PD [23].

### Personality traits and quality of life in Parkinson’s disease

It is clear that health-related and demographic variables significantly influence QoL in PwPD, but personality traits offer a deeper understanding of individual differences in coping and adaptation. Personality has been described in the literature using various theoretical models. One popular model is the five-factor model commonly called Big Five, which operationalizes personality traits through the dimensions of *neuroticism, extroversion, conscientiousness, openness to experience, and agreeableness* [24,25]. Its illustration of personality traits seeks to explain the differences between individuals in how they are likely to perceive, cope with and react to different events, illnesses or life circumstances [26]. *Neuroticism* is defined as emotional instability [24,25]. It describes the predisposition to negative emotions, such as anxiety, guilt, depression, and anger, and to unwarranted worry. Consequently, research has shown that individuals with elevated levels of neuroticism tend to utilize healthcare services more frequently [27], leading to a lower incidence of mortality among individuals with a high expression of this personality trait [28]. *Extraversion* refers to the patterns of activity and interpersonal behavior exhibited by individuals. It is also sometimes referred to as surgency [29]. *Conscientiousness* refers to the inclination to adhere to socially prescribed norms for impulse control, goal orientation, planning, and the ability to defer gratification [30]. Common terminology suggests that *openness* to experience relates to curiosity, an appreciation of aesthetics, and a willingness to explore new ideas without reluctance [31]. *Agreeableness* is the extent to which people try to come to good terms with other people [24,25].

#### Neuroticism.

A substantial body of evidence from both cross-sectional and longitudinal studies consistently demonstrates a negative correlation between neuroticism and QoL. This is evidenced by the findings of Dubayova et al. [16] and Pontone et al. [13]. This association may be attributed to the tendency of neuroticism to amplify perceived stressors, health-related anxieties, and depressive symptoms, which are prevalent in PwPD [32]. Furthermore, neuroticism is associated with maladaptive coping strategies, such as rumination, which exacerbate emotional distress and reduce overall well-being [33].

In PwPD, neuroticism intensifies the psychological burden of both motor and non-motor symptoms, including cognitive decline and depression [12,16]. Gale et al. [28] suggest that neuroticism’s focus on health anxiety may lead to overestimation of symptom severity, further reducing QoL. This dimension of personality may explain why PwPD report lower SRH, even when controlling for objective health measures [22].

#### Conscientiousness.

Conscientiousness positively influences QoL in PD by promoting self-discipline, goal orientation, and adherence to medical regimens. Studies suggest that individuals with higher conscientiousness demonstrate better medication adherence and resilience in coping with PD-related challenges [34,35]. These traits are critical in a disease that requires consistent management of motor and non-motor symptoms. In addition, conscientiousness is associated with better mental health outcomes and reduced functional impairment, indirectly improving QoL [22].

Conscientious individuals are also less likely to experience functional impairment and depressive symptoms, which are important mediators of QoL in PwPD [16,34]. In addition, they often employ adaptive coping mechanisms that enhance resilience in the face of disease challenges, contributing to sustained psychological well-being.

#### Openness.

The relationship between openness and QoL in PwPD is less well documented and often context dependent. Openness could offset some of the cognitive decline associated with PD [31]. Studies [36,37] found that openness promotes a sense of control and engagement, which may improve QoL in PwPD participating in novel therapies such as music or art therapy.

However, the influence of openness on QoL appears to be weaker than that of neuroticism or conscientiousness [34,38]. In longitudinal analyses, openness has shown modest positive effects, suggesting that its influence may be more pronounced in early stages of illness or under certain environmental conditions [35].

#### Other personality traits.

Extraversion and agreeableness have limited but positive effects on QoL in PwPD. Extraversion may buffer against social isolation, a common problem in PwPD [13]. Agreeableness may enhance social support networks and indirectly improve QoL [16]. However, these traits have less consistent and weaker associations with QoL compared to neuroticism and conscientiousness.

### Research contributions

No longitudinal studies have examined the predictive impact of specific personality traits on QoL in PwPD to date. However, understanding this link between personality and PD is of particular interest since PwPD suffer from reduced QoL stemming from both motor and nonmotor symptoms, both of which may be ameliorated by certain personality traits and their protective effect on health. This raises the question that we aim to address in this paper: Which personality traits have the strongest associations with QoL changes in PwPD when controlling for sociodemographic and health-related factors?

Thus, this study makes several important contributions to the existing body of research on QoL in PwPD. First, to expand existing research, this paper provides a longitudinal perspective by analyzing data from two waves of the SHARE dataset. While most previous studies have been cross-sectional, our approach allows for a deeper understanding of how personality traits predict changes in QoL over time. Second, the study specifically examines the Big Five personality traits and their independent and combined effects on QoL, providing nuanced insights into the interplay between personality traits and health outcomes in PwPD. Third, by integrating demographic, psychosocial and health-related variables as covariates, the paper offers a multidimensional analysis that accounts for the complex influences on QoL.

## Materials and methods

### Study design and population

Data from the Survey of Health, Aging and Retirement in Europe (SHARE), a longitudinal study of adults across 20 European countries and Israel, were utilized. The use of probability sampling ensured a nationally representative sample. As inclusion criteria for SHARE, the selected households had to have at least one person over the age of 50 who acted as the primary respondent. The survey was conducted through both computer-assisted personal interviews and a paper-pencil questionnaire. It covered several categories, including demographics, socio-cultural elements, functional ability, mental health, and other health-related indicators. The survey materials provide extensive details regarding the sampling design, data resources, and survey methods [39–44]. Data were extracted from the easySHARE dataset from waves 7 (2017) to 8 (2019) [45,46]. As the utilized questionnaires may vary between waves, we selected waves 7 and 8 due to the availability of the Big Five Inventory-10 (BFI), which is not available in all waves. To minimize the phenomenon of survivorship bias, adjustments have been carried out since wave 7 (from this wave onwards we obtain our data). Cleaning rules for the SHARE longitudinal sample are as follows: Households in which none of the eligible members has participated in three or more consecutive waves are dropped from the longitudinal sample. Non-participation may be due to non-contact, refusal, unknown address, or any other justified reason. In general, SHARE endeavors to identify and engage respondents (panelists) for interviews, and has achieved a high level of success, with over 80% of its respondents being panelists [47].

Survivorship bias arises whenever missing data occurs because of a non-random mechanism. Consequently, while the bias induced by demographic differences in follow-up survey participation may be mitigated through the application of post-stratification weighting for observed variables in accordance with population estimates [48],

The participants were selected based on the criteria outlined in question “Has a doctor ever told you that you had Parkinson’s disease?”. Furthermore, the data set was filtered to include only those with a complete QoL assessment and BFI. Our selected group was supplemented with the variables listed in the S1 File to account for relevant demographic and psychosocial factors in the analysis.

### Measures

For the purpose of clarity and replicability, we provide the variable names in the original dataset [49] in the S1 File.

### Dependent variable

The assessment of QoL is carried out through the CASP-12 (CASP). The CASP is a condensed iteration of the CASP-19, which assesses the quality of life (QoL) in older adults. The CASP-19 is anchored in a sociological conceptualization of QoL, drawing upon the “Theory of Human Need” [50], and comprises four sub-dimensions: Control, Autonomy, Self-realization, and Pleasure, which are then summed to produce a CASP score ranging from 12 to 48. A high score indicates a greater QoL [51]. The reliability coefficient is found to be satisfactory for the autonomy dimension with an average Cronbach`s alpha value of 0.71 and for the self-realization dimension with an average of 0.85. The internal consistency of the control dimension is satisfactory (average α = 0.73), and in the majority of countries, it is above or very close to the cut-off of 0.70. With respect to the pleasure dimension, the Cronbach’s alpha is, on average, satisfactory (average α = 0.74), and above.70 in the majority of countries [50].

**Fig 1:**
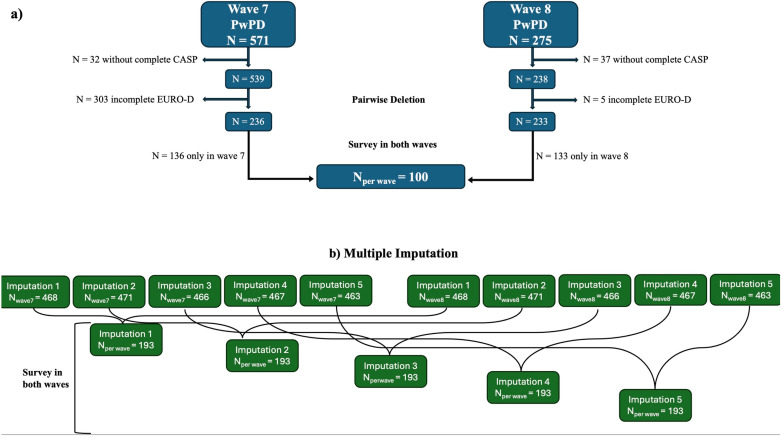
Sample size flow chart. (a) Flow chart of the sample size, (b) Multiple Imputation.

In total, 539 PwPD had a complete CASP at wave 7, and 238 participants had a complete CASP at wave 8. Following the application of pairwise deletion to filter for complete data, the cohort was reduced to 233 participants across Wave 8 and 236 across wave 7. Subsequently, the selection was further refined to include only participants who were surveyed in both waves, resulting in a final sample size of 100 PwPD (Fig 1a). Due to the high dropout rate, the results were controlled using multiple imputation as described in the Statistical Analysis section. Fig 1b illustrates how the sample size was determined.

### Covariates

We included a number of covariates to see whether the effect of personality persists when other known variables are included in the model. These covariates were based on existing literature on factors influencing QoL in PD and other chronic illnesses. These variables encompass a range of factors, including demographic characteristics, psychosocial factors, health-related factors, and personality traits [7,10–16]. The variables, their sources, and methods of assessment are outlined in the following sections

1. **Demographic Variables**
  - Age (derived from interviews): Recorded in years at the time of the interview
  - Sex (derived from interviews): Male or female
  - Education (self-reported): Measured as the total years of full-time schooling and vocational training
  - Marital Status (derived from interviews): Categorized as married/living together, registered partnership, divorced, widowed, or never married
2. **Personality Traits**
  - Big Five Inventory-10 (BFI) [52] (self-reported): To determine the participant’s individual personality expression across five dimensions (extraversion*,* agreeableness*,* conscientiousness*,* neuroticism*,* openness), the responses to two items within each dimension are averaged. A number of studies have demonstrated that the BFI is a reliable instrument. In one sample, the test-retest correlations were between r = 0.65 (Openness) and r = 0.79 (Extraversion) over a period of 6–8 weeks [52]. Similar outcomes were observed for the German BFI items in multiple studies [53]. The items’ internal consistencies are markedly inferior to the test-retest correlations. Nevertheless, the internal consistency underestimates the reliability for heterogeneous scales such as the BFI, where the items are designed to assess discrete aspects of the construct (Self-reported)
3. **Health-Related Variables**
  - Self-Rated Health (SRH) (self-reported): Assessed using a five-point Likert scale (excellent to poor) to gauge participants’ perception of their general health
  - Depressive Symptoms (self-reported): Measured using the EURO-D scale [54], comprising 12 binary items: sadness, pessimism, suicidal ideation, guilt, sleep disruptions, reduced interest in activities, irritability, decreased appetite, fatigue, impaired concentration, lack of pleasure, and tearfulness. The 12 EURO-D items were derived from the Geriatric Mental State [55]. Each symptom is assigned a score of either 0 or 1, with 1 consistently indicating a negative emotional state (i.e., 1 = higher degree of depression). The results of the 12 items are consolidated to obtain an overall score that ranges from 0 to 12 [54]. The EURO-D was specifically designed to assess depressive symptoms in European study populations; it does not have another name [54]. In the EURODEP study, the internal consistency of the EURO-D was moderately high, with a Cronbach’s alpha value ranging from 0.61 to 0.75 [54]. The evidence for internal consistency and construct validity of the EURO-D scale was reinforced following its utilisation in the 10-nation European SHARE study. It was demonstrated to be a hierarchical scale with analogous rank ordering of item calibration values across countries [56].
  - Number of Chronic Diseases (self-reported): Derived from participants’ responses to whether they had been diagnosed with specific chronic conditions, including PD
  - Mobility Index (self-reported):: Summed scores of difficulties in walking 100 meters, walking across a room, climbing several flights of stairs, and climbing one flight of stairs. The index ranges from 0 to 4. A higher mobility index indicates greater challenges in performing these activities and a lower level of mobility for the individual being evaluated.
4. **Functional Ability**
  - Activities of Daily Living (ADL) (derived from interviews): An index summarizing difficulties with basic tasks: dressing, bathing or showering, eating, cutting up food, walking across a room, and getting in or out of bed.
  - Instrumental Activities of Daily Living (IADL) (derived from interviews): An index of challenges in more complex tasks: telephone calls, taking medications, managing money, shopping for groceries, and preparing a hot meal.

The higher the index is, the more difficulties with the (I)ADLs. ADL and IADL range from 0 to 5.

1. **Cognitive Function**
  - Memory Function (Word Recall) (derived from interviews): Participants were asked to recall a list of words to measure cognitive performance, with scores ranging from 0 to 10.

### Statistical analysis

All data analyses were performed with IBM SPSS Statistics (version 29) and R (version 4.1.1). Based on the results of the Shapiro‒Wilk test, the parameters under investigation did not follow a normal distribution. Hence, for metric values, the median and interquartile range (IQR) are reported, while numerical and percentage forms are used for nominal values. Pairwise deletion was employed to manage any missing values. To reduce the amount of missing values, additional multiple imputation was performed using predictive mean matching with five imputed datasets. The imputation process was conducted using IBM SPSS Statistics software. The imputation targeted the EURO-D variable. Additionally, the CASP was included as second variable in the analysis, following the general strategy of incorporating the outcome variable to maintain associations between predictors and outcomes [57]. All analyses were performed on each imputed dataset and results were combined using standard Rubin rules [58]. This method has already been used for the SHARE datasets [59,60]. All statistical tests performed were two-tailed and at a significance level of 0.05.

To ascertain factors associated with QoL in each wave, we performed group comparisons (using Wilcoxon and Chi^2^ tests) and linear regression analyses with stepwise backward selection for the analysis with covariates and Akaike’s information criterion as the selection criterion. This ensured cross-sectional identification of QoL factors. The Durbin Watson Test indicated that autocorrelation was not an issue in the model, with a value between 1 and 2. Additionally, there was no evidence of multicollinearity, as indicated by Variance Inflation Factor values between 1 and 2 in all regressions of the respective waves.

Generalized estimating equations (GEEs) were employed to investigate the potential longitudinal impact of personality traits on the QoL metric. The GEE method is a longitudinal approach that aims to estimate mean relationships while addressing nuisance covariances individually. The technique aims to enhance estimation precision and ensure valid significance testing by integrating marginal effects. GEE computes two equations, one for mean relations and one for covariance structure [61]. The GEE modeled utilizing a Poisson distribution and an independent correlation structure. The best fit was established through the quasi-likelihood under independence model criterion [62]. The GEE-based tests utilizing empirical sandwich estimator criteria have been demonstrated to exhibit minimal sensitivity to misspecification of the covariance structure models. The application of a random coefficients yielded robust tests with competitive power across all conditions examined [63]. This was employed in our analysis.

In accordance with the prevailing guideline for regression analysis, including GEE, a minimum of 10–15 observations per explanatory variable is typically advised. Consequently, a sample size of 100 allows for the inclusion of up to 10 variables in the model [64].

The rationale behind the suitability of this approach is as follows: GEE is designed for longitudinal or clustered data, which aligns with the panel nature of the data in the second document (e.g., SHARE waves). It accounts for within-subject correlations and can model the mean quality of life trajectory over time. The best use of this method is when the focus is on estimating population-averaged effects of personality traits on QoL. The advantages of this method are as follows: it handles correlated outcomes (e.g., repeated measures on the same individuals); it has flexible correlation structures (e.g., independence, exchangeable); and it is robust to misspecification of the correlation structure [65–67].

To ascertain the reliability of the estimated standard errors of the GEE model, a cluster-based bootstrapping procedure was employed. In this approach, complete clusters were randomly resampled with backspacing to account for the dependency between repeated measurements within the clusters. For each bootstrap repetition, the GEE model was adjusted while retaining the original model structure. A total of 1000 bootstrap repetitions were performed to obtain robust standard errors. The robust standard errors were calculated by the standard deviation of the estimated coefficients from the bootstrap replicates and then compared with the original standard errors.

## Results

**Diagram 1:**
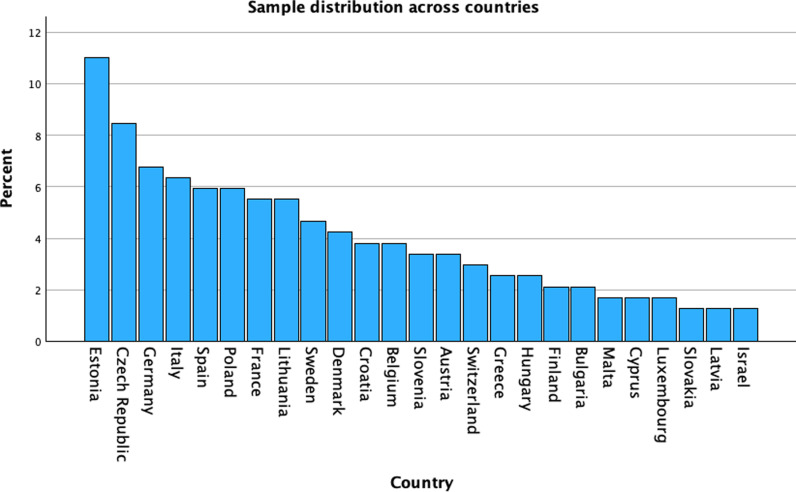
Sample distribution across countries.

Table 1 shows the descriptive statistics of the PwPD for the two waves. The median age of participants in Wave 7 was 74 years, with a slight increase to 77 years in Wave 8. Neuroticism was the most prominent personality trait, followed by agreeableness, while openness and conscientiousness showed consistent moderate levels. Overall, 53.9% (n = 303 in wave 7) and 56.7% (n = 139 in wave 8) of participants were male. The majority of participants of both waves originated from Estonia (11.3%), with the Czech Republic and Germany accounting for 8.5% and 7.8% of participants, respectively (Diagram 1). From wave 7 to wave 8, QoL, depressive symptoms, ADL, IADL, recall, and mobility changed, albeit with small effect sizes. The personality trait distribution of each wave, based on age- and sex-specific cutoffs, indicated that PwPD displayed the highest levels in the traits neuroticism and agreeableness. Owing to a high dropout rate between the initial and final cohorts, especially in wave 7, which were used for longitudinal analysis, we conducted an analysis to determine whether any specific personality traits were excluded, potentially impacting the results. However, we found no evidence of this (S2 Table).

**Table 1:** Descriptive statistics.

		wave 7	wave 8	group comparison	
		median (IQR)/	median (IQR)/	Wilcoxon-test (R^2^)	p
		mean (SD)	mean (SD)		
Age in years		74 (70-80)/	77 (70-80)/	**0.68**	**<0.001**
		74 (7)	77 (7)		
Education in years		12 (7-13)/	12 (8-14)/	1.00	1.00
		12 (5)	12 (5)		
CASP		34 (27-36)/	34 (28-37)/	**0.03**	**0.01**
		34 (6)	33 (7)		
EURO-D		3 (2-6)/	3 (2-6)/	**0.11**	**0.01**
		3 (3)	4 (3)		
BFI – Extraversion		3 (3-4)/	3 (3-4)/	–	0.63
		3 (1)	3 (1)		
BFI – Agreeableness		4 (3-5)/	4 (3-4)/	–	0.23
		4 (1)	4 (1)		
BFI – Conscientiousness		4 (4-5)/	4 (4-5)/	–	0.37
		4 (1)	4 (1)		
BFI – Neuroticism		3 (2-4)/	3(2-4)/	–	0.46
		3 (1)	3 (1)		
BFI – Openness		3 (3-4)/	3 (3-4)/	–	0.38
		3 (1)	3 (1)		
ADL		0 (0-2)/	1 (0-2)/	**0.08**	**<0.001**
		1 (1)	1 (2)		
IADL		0 (0-2)/	0 (0-2)/	**0.14**	**<0.001**
		1 (1)	1 (2)		
Recall		5 (3-5)/	4 (3-6)/	**0.03**	**0.01**
		5 (2)	4 (2)		
Mobility index		1 (0-3)/	2 (0-3)/	**0.02**	**0.03**
		1 (1)	2 (1)		
		**n (%)**	**n (%)**	**Chi** ^**2**^ **-Test (Cramers V)**	**p**
Sex	male	303 (53.9%)	139 (56.7%)	–	0.46
	female	259 (46.1%)	106 (43.3%)		
Marital status	married, living together	364 (64.9%)	154 (63.1%)	–	0.84
	registered partnership	7 (1.2%)	3 (1.2%)		
	married, living separated	6 (1.1%)	3 (1.2%)		
	never married	22 (3.9%)	15 (6.1%)		
	divorced	26 (4.6%)	12 (4.9%)		
	widowed	136 (24.2%)	57 (23.4%)		
SRH	excellent	3 (0.5%)	1 (0.4%)	–	0.27
	very good	9 (1.6%)	7 (2.9%)		
	good	76 (13.5%)	41 (16.7%)		
	fair	217 (38.6%)	102 (41.6%)		
	poor	257 (45.7%)	94 (38.4%)		

ADL, limitations in activities of daily living; BFI, big five inventory; CASP, Control, Autonomy, Self-realization, Pleasure (QoL) Score; EURO-D, depressive symptoms questionnaire; IADL, limitations in instrumental activities of daily living; IQR, interquartile range; SD, standard deviation; SRH, self-rated health

### Personality traits cross-sectionally associated with QoL

First, we identified factors that were cross-sectionally associated with QoL in both waves. For this purpose, we calculated Spearman’s correlations (S3 Table) and we built several regression models. The correlations showed that QoL at wave 7 was significantly correlated with all selected variables, especially with all personality traits. At wave 8, however, there was no significant correlation between QoL and the personal traits of conscientiousness and openness.

In an initial model, we only included the personality traits as covariates. In both waves, neuroticism showed a strong negative association with QoL (β = -0.33, p < 0.001 in Wave 7; β = -0.28, p < 0.001 in Wave 8). Extraversion, agreeableness, and conscientiousness had weaker but statistically significant positive effects in Wave 7 (S4 and S5 Tables).

In a second step, to assess the contribution of personality traits in addition to other variables known to influence QoL, we additionally controlled for several cofactors with backward selection. In wave 7, only neuroticism (β = -0.2, p < 0.001) and openness (β = 0.12, p = 0.02) remained in the model alongside depressive symptoms (β = -0.34, p < 0.001), mobility limitations (β = -0.23, p < 0.001), and SRH (β = -0.13, p = 0.02). These variables explained 46% of the QoL variance (S6 Table). In wave 8, agreeableness (β = 0.10, p = 0.06) and extraversion (β = 0.10, p = 0.06) were associated with QoL when adjustment for covariates (adjusted R² = 0.46) (S7 Table).

These findings were confirmed using data from multiple imputation. In Wave 7, the results remained consistent with the imputed data: Neuroticism (β between -0.21 and -0.18, p < 0.001) and openness (β between 0.07 and 0.10, p between 0.002 and 0.03) were the only personality traits that significantly influenced QoL across all five imputations. Additionally, extraversion (β between 0.07 and 0.08, p between 0.01 and 0.03) was significant in imputations one, two, and four, while conscientiousness (β = 0.07, p = 0.04) showed significance in imputations one and two. Other significant covariates varied only by country and age in imputation three (p = 0.06). Across all imputations, country also had a significant effect on QoL (β between -0.15 and -0.13, p < 0.001) (S8 Table). In Wave 8, the results from imputation one was similar (Agreeableness and extraversion: β = 0.10, p > 0.05). However, the other imputations showed inconsistent effects for agreeableness and extraversion. In imputations two and three, agreeableness had a significant effect on QoL (β between 0.11 and 0.12, p = 0.03), whereas in imputations four and five, extraversion was a significant predictor of QoL (β = 0.11, p between 0.03 and 0.04). The other covariates remained largely consistent, except in imputation two, where recall replaced extraversion but had no significant effect (β = 0.10, p = 0.10) (S9 Table).

### Personality traits longitudinally associated with QoL

To assess the causal relationship between QoL and personality, we next assessed whether baseline personality traits influence future QoL. For this purpose, GEEs were calculated with and without adjustment for covariates. The variables that demonstrated a notable impact in the cross-sectional linear regressions (S6–S9 Table) were included as covariates. When selecting the BFI traits, all except agreeableness were included, as this did not exhibit significant values in any of the four regressions (S4 and S5 Table). Without adjustment for covariates, neuroticism (β = -0.09, p < 0.001) and conscientiousness (β = 0.07, p = 0.001) had a small predictive effect on QoL (Table 2). With adjustment for covariates, neuroticism retained its predictive power for QoL decline over time (β = -0.03, p = 0.007), while openness showed a smaller and positive effect (β = 0.02, p = 0.008). Other predictors of QoL were depressive symptoms (β = -0.02, p = 0.001) and mobility limitations (β = -0.02, p = 0.02) (Table 3). Based on the small difference (<10%) between the SE of the GEEs and the bootstraps, it can be assumed that the GEEs are robust (Tables 2–5).

**Table 2:** Simple GEE with personality traits.

Variable	Beta (SE)	HR (95% CI)	p	SE from bootstrap
Intercept	3.49 (0.12)	–	**< 0.001**	0.04
BFI – Neuroticism	-0.09 (0.02)	0.91 (0.88, 0.95)	**< 0.001**	0.01
BFI – Openness	0.03 (0.02)	1.03 (0.99, 1.06)	0.13	0.01
BFI – Conscientiousness	0.07 (0.02)	1.08 (1.03, 1.12)	**0.001**	0.01
BFI – Extraversion	-0.02 (0.02)	0.98 (0.94, 1.01)	0.21	0.01

n = 100.

BFI, big five inventory; CI, confidence interval; HR, Hazard ratio; p, significance; SE, standard error.

**Table 3:** GEE with adjustment for covariates.

Variable	Beta (SE)	HR (95% CI)	p	SE from bootstrap
Intercept	4.12 (0.21)	–	**< 0.001**	0.13
BFI – Neuroticism	-0.03 (0.01)	0.97 (0.96, 0.99)	**0.007**	0.01
BFI – Openness	0.02 (0.01)	1.03 (1.01, 1.04)	**0.008**	0.01
BFI – Conscientiousness	0.01 (0.01)	1.01 (0.99, 1.04)	0.24	0.01
BFI – Extraversion	0.00 (0.01)	1.00 (0.98, 1.03)	0.76	0.01
SRH	-0.05 (0.03)	0.95 (0.90, 1.00)	0.07	0.02
EURO-D	-0.03 (0.00)	0.97 (0.96, 0.98)	**0.001**	0.01
IADL	-0.01 (0.01)	0.99 (0.98, 1.01)	0.39	0.01
Recall	0.01 (0.01)	1.01 (1.00, 1.02)	0.07	0.02
Mobility	-0.02 (0.01)	0.98 (0.96, 1.00)	**0.02**	0.01

n = 100.

ADL, limitations in activities of daily living; BFI, big five inventory; CI, confidence interval; EURO-D, depressive symptoms questionnaire; HR, Hazard ratio; IADL, limitations in instrumental activities of daily living; SE, standard error; SRH, self-rated health.

**Table 4:** GEE with Imputed Data (Imputation 1).

Variable	Beta (SE)	HR (95% CI)	p	SE from bootstrap
Intercept	3.52 (0.08)	–	**< 0.001**	0.06
BFI – Neuroticism	-0.05 (0.01)	0.95 (0.93, 0.97)	**< 0.001**	0.01
BFI – Openness	0.01 (0.01)	1.01 (0.99, 1.03)	0.43	0.01
BFI – Extraversion	0.00 (0.01)	1.00 (0.98, 1.03)	0.81	0.01
BFI – Conscientiousness	0.03 (0.02)	1.03 (1.00, 1.06)	0.09	0.01

n = 193.

BFI, big five inventory; CI, confidence interval; HR, Hazard ratio; p, significance; SE, standard error.

**Table 5:** GEE Analysis with Imputed Data and Covariate Adjustment (Imputation 1).

Variable	Beta (SE)	HR (95% CI)	p	SE from bootstrap
Intercept	3.57 (0.06)	–	**< 0.001**	0.09
BFI – Neuroticism	-0.02 (0.01)	0.98 (0.96, 0.99)	**0.003**	0.01
BFI – Openness	0.01 (0.01)	1.01 (1.00, 1.03)	0.16	0.01
BFI – Extraversion	0.01 (0.01)	1.01 (0.99, 1.03)	0.23	0.01
BFI – Conscientiousness	0.01 (0.01)	1.01 (0.99, 1.03)	0.26	0.01
SRH	-0.06 (0.04)	0.94 (0.87, 1.01)	0.09	0.02
EURO-D	-0.03 (0.00)	0.97 (0.97, 0.98)	**< 0.001**	0.01
IADL	-0.01 (0.01)	0.99 (0.98, 1.00)	0.17	0.08
Recall	0.01 (0.00)	1.01 (1.00, 1.02)	**0.02**	0.01
Mobility	-0.02 (0.01)	0.98 (0.97, 1.00)	**0.02**	0.01

n = 193.

BFI, big five inventory; CI, confidence interval; EURO-D, depressive symptoms questionnaire; IADL, instrumental activities of daily living; HR, Hazard ratio; p, significance; SE, standard error; SRH, self-rated health.

These findings were confirmed using multiple imputation, with all five datasets yielding consistent results. Therefore, we did not include separate GEE tables for each analysis but instead provide an exemplary analysis using Imputation 1 (Tables 4 and 5).

Without covariate adjustment, neuroticism remained significant (β = -0.05, p < 0.001), while conscientiousness did not (p = 0.09) (Table 4). After adjustment, neuroticism was the only personality trait to retain its predictive power for QoL decline over time (β = -0.03, p = 0.003).

The other covariates also showed the same results: mobility limitations (β = -0.02, p = 0.02) and depressive symptoms remained predictors (β = -0.03, p < 0.001). Additionally, cognitive function (β = 0.01, p = 0.02) had a small but significant impact on QoL (Table 5).

These findings highlight the pivotal role of neuroticism in QoL, with depressive symptoms and mobility limitations serving to further compound its impact.

## Discussion

This study offers several significant contributions to the understanding of QoL determinants in PwPD. The study provided longitudinal insights on the relationship between personality and QoL. By leveraging longitudinal data, it extends the cross-sectional evidence base, demonstrating the predictive impact of specific personality traits – particularly neuroticism –on QoL over time. The results emphasized the interplay between personality traits and health-related variables, such as depressive symptoms and SRH, highlighting the multifaceted influences on QoL. Our findings advocate for the integration of personality assessments into clinical practice to inform tailored interventions aimed at enhancing resilience and mitigating negative QoL impacts in PwPD.

### Neuroticism as a key predictor

Our findings corroborate those of prior research which identifies neuroticism as a consistent negative predictor of QoL across various chronic conditions [17], including heart failure [68], multiple sclerosis [69], and epilepsy [70]. In PwPD, our findings confirm that neuroticism exerts a robust and independent influence on QoL both cross-sectionally and longitudinally. These results corroborate earlier cross-sectional findings [13–16], The heightened vulnerability of individuals with high neuroticism to negative emotions and ineffective coping strategies [71–73] is likely to exacerbate the progressive and fluctuating nature of PD. This is supported by neurochemical links such as the 5-HTTLPR polymorphism in serotonin regulation [74].

### The role of conscientiousness and openness

In contrast, conscientiousness was identified as a factor with a positive association with QoL, particularly in unadjusted longitudinal analyses. This is consistent with studies in both PwPD [34] and the general population [35], which emphasized the protective effects of organized and goal-directed behaviors [13]. This could increase patients’ well-being due to their efficient, hardworking, and organized nature, which in turn increases their ability to achieve personal goals and improve their overall QoL [75,76]. Especially in PD, which requires adherence to a complex and strict regimen of medication and therapy, a conscientious mindset may help with the management of this extensive illness.

In the longitudinal analysis, openness was found to have a minor but positive predictive effect on QoL when controlling for other factors. This suggests that PwPD with higher openness scores may experience slightly better QoL over time, potentially due to their adaptability and engagement in life-enhancing activities [31]. This finding is consistent with those observed in other chronic conditions, where openness is often associated with psychological resilience, though not as strongly as other traits [35].

### The role of depressive symptoms and SRH

Depressive symptoms significantly impact QoL, aligning with prior findings [22,32,77]. The bidirectional relationship between personality traits and depressive symptoms [78] suggests that addressing subthreshold depression in PwPD could mitigate neuroticism’s adverse effects on QoL. Additionally, SRH emerged as a key QoL factor [22,32]. potentially shaped by health-related anxieties linked to neuroticism. Neuroticism, marked by emotional instability and a focus on negative outcomes, likely amplifies health concerns, lowering SRH and QoL [22,32,33]. In PwPD, where symptom progression varies, these tendencies may heighten the perceived impact of health challenges.

In comparison to cross-sectional studies, our longitudinal approach demonstrates that the impact of neuroticism remains consistent over time, whereas the effects of traits such as conscientiousness and openness appear to be more context dependent. In contrast to broader cohorts of individuals with chronic diseases, those with PD face distinctive challenges [6], including fluctuating motor and non-motor symptoms [5], which may intensify the psychological impact of traits such as neuroticism. Furthermore, while previous research has emphasized the significance of clinical factors such as mobility limitations, age, depressive symptoms, and limitations in ADL [7,10–12] our study demonstrates that personality traits independently contribute to QoL variance even after controlling for these variables. This highlights the necessity for a comprehensive approach to the management of PD that considers psychological and personality dimensions in addition to traditional medical care [10].

Furthermore, while previous research has emphasized the significance of clinical factors such as mobility limitations and depressive symptoms [7,10], our study demonstrates that personality traits independently contribute to QoL variance even after controlling for these variables. This highlights the necessity for a comprehensive approach to the management of PD that considers psychological and personality dimensions in addition to traditional medical care.

The study has limitations. One limitation is the lack of differentiation between idiopathic PD and secondary Parkinson’s syndrome. Additionally, the diagnosis of PD itself depends on self-report. The dataset only includes community-dwelling individuals, suggesting that the examined sample has a mainly positive general state of health. As a result, conclusions concerning individuals with severe health restrictions are limited. A further limitation is the sample size. The fact that a large number of participants had to be excluded because they were not interviewed in both waves should be viewed critically. As detailed in the methodology section, this was due to the selection criteria and the requirement that participants in both waves completed all the necessary questionnaires. Regardless of the analytical techniques employed, this results in low power [79], unstable estimates [64], an increased probability of overfitting [80], and a lack of generalizability [79]. We attempted to minimize this by reducing the number of variables included in the analyses to a minimum and added a bootstrap. The bootstrap method, with minor discrepancies between the standard errors allows for the generation of robust results. To enhance the robustness of our findings and minimize potential biases, we performed multiple imputations to validate the results obtained through pairwise deletion. Our key conclusions remained consistent across both approaches, supporting the robustness of our methodology despite the high dropout rate. This consistency reinforces the reliability of our findings and confirms that missing data did not significantly distort the overall results. Nevertheless, some inconsistencies emerged across the imputation datasets in the cross-sectional analyses. While the primary statements regarding personality traits remained stable (In wave 7, neuroticism and openness influenced QoL in both analyses with covariates. In wave 8 In wave 8, agreeableness and extraversion were associated with QoL, with significance varying across imputations.), these inconsistencies reduce the quality of the cross-sectional analyses. This limitation should be considered when interpreting the cross-sectional findings, as it may introduce variability and diminish the robustness of the results. Several variables used in the analysis depend on self-reports, namely, QoL, depressive symptoms, SRH, and daily activities, suggesting that the given responses might lack impartiality. The use of self-reported data presents issues such as socially desirable responses and sampling bias [81]. However, according to existing studies [82], self-reporting remains a valuable source of information. In addition, all self-report instruments employed during the data collection process are validated and routinely utilized. Moreover, SHARE selected a standardized computer-assisted personal interviewing data collection method to mitigate the possibility of social desirability response bias. As QoL is a complex and diverse construct, it is influenced by a multitude of variables which were not exhaustively included in the present study. Therefore, it is possible that the effect of personality traits may differ depending on the included covariates. Future research should aim to assess the relative contribution of personality traits in comparison with other covariates in greater depth.

The findings make notable theoretical contributions by extending our understanding of the interplay between personality and QoL. In particular, the study highlights the critical role of neuroticism in shaping negative health perceptions. Furthermore, the integration of personality and disease-specific factors bridges psychological and neurological research and advances theoretical models of resilience and vulnerability in chronic illness.

The study also has important practical implications for clinical care and policy development. Personality assessments can help identify individuals at higher risk of poor quality of life, particularly those with high levels of neuroticism. These findings can inform personalised interventions, such as cognitive behavioural therapy or mindfulness-based stress reduction, aimed at improving emotional regulation and psychological resilience. Policy makers and healthcare providers can use these findings to design comprehensive care models that address the physical, emotional and psychological needs of PwPD.

## Conclusions

This study highlights the significant influence of personality traits, particularly neuroticism, on the QoL of PwPD. By integrating personality assessments and addressing modifiable health-related factors, care strategies can be refined to better support the psychological and emotional needs of PwPD, ultimately improving long-term outcomes. Our findings contribute significantly to the theoretical understanding of how personality traits interact with QoL longitudinally, emphasizing neuroticism’s pivotal role in shaping negative health perceptions. Moreover, the integration of personality and disease-specific factors bridges the gap between psychological and neurological research, advancing theoretical models of resilience and vulnerability in chronic illness. Ultimately, this study not only enhances practical care approaches but also enriches theoretical models by demonstrating the intricate interplay between personality and chronic disease management.

To build on these insights, future research should focus on large-scale, prospective longitudinal studies and explore targeted interventions to manage neuroticism as part of a holistic approach to PD treatment.

## Supporting information

S1 File Covariates overview. (DOCX)

S1 Table Comparison between the drop-out cohort and final cohort in wave 7. (DOCX)

S2 Table Spearman’s Correlation. (DOCX)

S3 Table Linear regression in wave 7 with BFI. (DOCX)

S4 Table Linear regression in wave 8 with BFI. (DOCX)

S5 Table Linear regression in wave 7 with covariates. (DOCX)

S6 Table Linear regression in wave 8 with covariates. (DOCX)

S7 Table Linear Regression with Imputed Data (Wave 7) and Covariates. (DOCX)

S8 Table Linear Regression with Imputed Data (Wave 8) and Covariates. (DOCX)
