# Supplementary material for: The influence of the Big Five inventory on quality of life in people with Parkinson’s disease aged 50 and above: A Longitudinal Analysis from the Survey of Health, Aging and Retirement in Europe (SHARE)
Source: PLoS One. 2025 May 30;20(5):e0322089. doi: 10.1371/journal.pone.0322089 (PMC12124528; doi:10.1371/journal.pone.0322089)
Supplement: S1 File — (DOCX) [file pone.0322089.s001.docx]

**S1 File. Covariates overview**

**1. Demographic Variables**

- Sex *(female),*
- age at interview in years *(age),*
- number of chronic diseases (*chronic_mod)*
- marital status *(mar_stat)*
- Education *(eduyears_mod)*

**2. Personality Traits**

- Big Five Inventory (BFI) across five dimensions (extraversion *[bfi10_extra_mod],* agreeableness *[bfi10_agree_mod],* conscientiousness *[bfi10_consc_mod],* neuroticism *[bfi10_neuro_mod],* openness *[bfi10_open_mod]*),

**3. Health-Related Variables**

- Self-rated health (*sphus*)
- Depressive symptoms as measured by the Centre for EURO-D (1) *[eurod]*
- number of chronic diseases (*chronic_mod)*
- mobility index *(mobilityind)*

**4. Functional Ability**

- ADLs *(adla) and* IADLs *(iadlza)*

**5. Cognitive Function**

- Recall of words *(recall_1)*
