# Supplementary material for: The influence of the Big Five inventory on quality of life in people with Parkinson’s disease aged 50 and above: A Longitudinal Analysis from the Survey of Health, Aging and Retirement in Europe (SHARE)
Source: PLoS One. 2025 May 30;20(5):e0322089. doi: 10.1371/journal.pone.0322089 (PMC12124528; doi:10.1371/journal.pone.0322089)
Supplement: S1 Table — (DOCX) [file pone.0322089.s002.docx]

**S2 Table. Comparison between the drop-out cohort and final cohort in wave 7**

| Wave 7 |  | **Median** | **Mean** | **SD** | **IQR** |
| --- | --- | --- | --- | --- | --- |
| Drop-out | BFI – Extraversion | 3.0 | 3.3 | 1.0 | 1.5 |
|  | BFI – Agreeableness | 3.5 | 3.7 | 0.9 | 1.5 |
|  | BFI – Conscientiousness | 4.0 | 3.9 | 0.9 | 1.0 |
|  | BFI – Neuroticism | 3.0 | 3.0 | 1.1 | 1.5 |
|  | BFI – Openness | 3.0 | 3.1 | 1.0 | 1.5 |
| Final cohort | BFI – Extraversion | 3.0 | 3.3 | 0.9 | 1.4 |
|  | BFI – Agreeableness | 4.0 | 3.9 | 0.8 | 1.0 |
|  | BFI – Conscientiousness | 4.0 | 3.9 | 0.8 | 1.0 |
|  | BFI – Neuroticism | 3.0 | 3.0 | 0.9 | 1.4 |
|  | BFI – Openness | 3.0 | 3.3 | 1.0 | 1.0 |

Note: BFI = Big Five Inventory, SD = standard deviation; IQR = Interquartile range
