# Supplementary material for: The influence of the Big Five inventory on quality of life in people with Parkinson’s disease aged 50 and above: A Longitudinal Analysis from the Survey of Health, Aging and Retirement in Europe (SHARE)
Source: PLoS One. 2025 May 30;20(5):e0322089. doi: 10.1371/journal.pone.0322089 (PMC12124528; doi:10.1371/journal.pone.0322089)
Supplement: S2 Table — (DOCX) [file pone.0322089.s003.docx]

**S3 Table. Spearman Correlation**

|  |  | **CASP (wave 7)** | **CASP (wave 8)** |
| --- | --- | --- | --- |
| Age in years | Correlation Coefficient | -0.18 | -0.20 |
|  | p | **< 0.001** | **< 0.001** |
|  | n | 562 | 333 |
| Education in years | Correlation Coefficient | 0.19 | 0.16 |
|  | p | **< 0.001** | **0.007** |
|  | n | 500 | 292 |
| CASP | Correlation Coefficient | 1.000 | 1.000 |
|  | p | . | . |
|  | n | 539 | 238 |
| EURO-D | Correlation Coefficient | -0.55 | -0.57 |
|  | p | **< 0.001** | **< 0.001** |
|  | n | 236 | 233 |
| BFI – Extraversion | Correlation Coefficient | 0.16 | 0.15 |
|  | p | **< 0.001** | **0.008** |
|  | n | 539 | 238 |
| BFI – Agreeableness | Correlation Coefficient | 0.11 | 0.19 |
|  | p | **0.008** | **< 0.001** |
|  | n | 539 | 238 |
| BFI – Conscientiousness | Correlation Coefficient | 0.18 | 0.08 |
|  | p | **< 0.001** | 0.173 |
|  | n | 539 | 238 |
| BFI – Neuroticism | Correlation Coefficient | -0.35 | -0.34 |
|  | p | **< 0.001** | **< 0.001** |
|  | n | 539 | 283 |
| BFI – Openness | Correlation Coefficient | 0.16 | 0.1 |
|  | p | **< 0.001** | 0.067 |
|  | n | 539 | 238 |
| ADL | Correlation Coefficient | -0.38 | -0.35 |
|  | p | **< 0.001** | **< 0.001** |
|  | n | 560 | 333 |
| IADL | Correlation Coefficient | -0.4 | -0.41 |
|  | p | **< 0.001** | **< 0.001** |
|  | n | 560 | 333 |
| Recall | Correlation Coefficient | 0.351 | 0.332 |
|  | p | **< 0.001** | **< 0.001** |
|  | n | 542 | 328 |
| Mobility index | Correlation Coefficient | -0.5 | -0.47 |
|  | p | **< 0.001** | **< 0.001** |
|  | n | 559 | 333 |
| Sex | Correlation Coefficient | - 0.16 | -0.11 |
|  | p | **< 0.001** | **0.04** |
|  | n | 562 | 333 |
| Marital status | Correlation Coefficient | -0.15 | -0.27 |
|  | p | **< 0.001** | **< 0.001** |
|  | n | 561 | 332 |
| SRH | Correlation Coefficient | -0.41 | -0.47 |
|  | p | **< 0.001** | **< 0.001** |
|  | n | 562 | 333 |

Note: ADL = limitations in activities of daily living; BFI = Big Five Inventory; BMI = body mass index; CASP = Control, Autonomy, Self-realization, Pleasure (QoL) Score; EURO-D = depressive symptoms questionnaire; IADL = limitations in instrumental activities of daily living; SRH = self-rated health
