# Supplementary material for: The influence of the Big Five inventory on quality of life in people with Parkinson’s disease aged 50 and above: A Longitudinal Analysis from the Survey of Health, Aging and Retirement in Europe (SHARE)
Source: PLoS One. 2025 May 30;20(5):e0322089. doi: 10.1371/journal.pone.0322089 (PMC12124528; doi:10.1371/journal.pone.0322089)
Supplement: S3 Table — (DOCX) [file pone.0322089.s004.docx]

**S4 Table. Linear regression in wave 7 with BFI**

| Model | B | SE | Beta | t | p | 95.0% CI for B | |
| --- | --- | --- | --- | --- | --- | --- | --- |
|  |  |  |  |  |  | Lower | Upper |
| Constant | 26.92 | 2.08 |  | 12.96 | **< 0.001** | 22.84 | 30.99 |
| BFI – Extraversion | 0.54 | 0.27 | 0.08 | 2.02 | **0.04** | 0.01 | 1.06 |
| BFI – Agreeableness | 0.59 | 0.30 | 0.08 | 1.95 | 0.05 | -0.01 | 1.18 |
| BFI – Conscientiousness | 1.13 | 0.29 | 0.15 | 3.93 | **< 0.001** | 0.57 | 1.69 |
| BFI – Neuroticism | -2.07 | 0.25 | -0.33 | -8.41 | **< 0.001** | -2.56 | -1.59 |
| BFI – Openness | 0.93 | 0.25 | 0.14 | 3.67 | **< 0.001** | 0.43 | 1.43 |

Dependent Variable: CASP, n = 539

adjusted R^2^ = 0.2, F(5, 533) = 27.14, p < 0.001; Durbin-Watson = 1.76

Note: BFI = Big Five Inventory; CASP = Control, Autonomy, Self-realization, Pleasure (QoL) Score; CI = Confidence Interval; SE = Standard Error
