# Supplementary material for: The influence of the Big Five inventory on quality of life in people with Parkinson’s disease aged 50 and above: A Longitudinal Analysis from the Survey of Health, Aging and Retirement in Europe (SHARE)
Source: PLoS One. 2025 May 30;20(5):e0322089. doi: 10.1371/journal.pone.0322089 (PMC12124528; doi:10.1371/journal.pone.0322089)
Supplement: S4 Table — (DOCX) [file pone.0322089.s005.docx]

**S5 Table. Linear regression in wave 8 with BFI**

| Model | B | SE | Beta | t | p | 95.0% CI for B | |
| --- | --- | --- | --- | --- | --- | --- | --- |
|  |  |  |  |  |  | Lower | Upper |
| Constant | 31.12 | 3.32 |  | 9.38 | **< 0.001** | 24.58 | 37.66 |
| BFI – Extraversion | 0.33 | 0.42 | 0.05 | 0.77 | 0.44 | -0.51 | 1.16 |
| BFI – Agreeableness | 0.51 | 0.47 | 0.07 | 1.08 | 0.28 | -0.42 | 1.44 |
| BFI – Conscientiousness | 0.78 | 0.48 | 0.11 | 1.62 | 0.11 | -0.17 | 1.74 |
| BFI – Neuroticism | -1.79 | 0.41 | -0.28 | -4.33 | **< 0.001** | -2.6 | -0.97 |
| BFI – Openness | 0.26 | 0.44 | 0.04 | 0.60 | 0.55 | -0.61 | 1.13 |

Dependent Variable: CASP, n = 238

adjusted R^2^ = 0.1, F(5, 232) = 6.23, p < 0.001; Durbin-Watson = 1.69

Note: BFI = Big Five Inventory; CASP = Control, Autonomy, Self-realization, Pleasure (QoL) Score; CI = Confidence Interval; SE = Standard Error
