# Supplementary material for: The influence of the Big Five inventory on quality of life in people with Parkinson’s disease aged 50 and above: A Longitudinal Analysis from the Survey of Health, Aging and Retirement in Europe (SHARE)
Source: PLoS One. 2025 May 30;20(5):e0322089. doi: 10.1371/journal.pone.0322089 (PMC12124528; doi:10.1371/journal.pone.0322089)
Supplement: S5 Table — (DOCX) [file pone.0322089.s006.docx]

**S6 Table. Linear regression in wave 7 with covariates**

| Model | B | SE | Beta | t | p | 95.0% CI for B | |
| --- | --- | --- | --- | --- | --- | --- | --- |
|  |  |  |  |  |  | Lower | Upper |
| Constant | 42.68 | 2.16 |  | 19.78 | **<0.001** | 38.43 | 46.93 |
| BFI – Neuroticism | -1.23 | 0.30 | -0.20 | -4.06 | **<0.001** | -1.83 | -0.63 |
| BFI – Openness | 0.76 | 0.31 | 0.12 | 2.45 | **0.02** | 0.15 | 1.38 |
| Country | -0.05 | 0.03 | -0.08 | -1.70 | 0.09 | -0.11 | 0.01 |
| SRH | -0.97 | 0.43 | -0.13 | -2.29 | **0.02** | -1.81 | -0.14 |
| EURO-D | -0.78 | 0.13 | -0.34 | -6.04 | **<0.001** | -1.04 | -0.53 |
| Mobility | -1.04 | 0.26 | -0.23 | -4.02 | **<0.001** | -1.55 | -0.53 |
| Recall | 0.30 | 0.17 | 0.10 | 1.77 | 0.08 | -0.03 | 0.64 |

Dependent Variable: CASP, n = 212

adjusted R^2^ = 0.46, F(7,227) = 28.91, p < 0.001; Durbin-Watson = 1.69

Note: BFI = Big Five Inventory; CASP = Control, Autonomy, Self-realization, Pleasure (QoL) Score; CI = Confidence Interval; EURO-D = depressive symptoms questionnaire; SE = Standard Error; SRH = self-rated health
