# Supplementary material for: The influence of the Big Five inventory on quality of life in people with Parkinson’s disease aged 50 and above: A Longitudinal Analysis from the Survey of Health, Aging and Retirement in Europe (SHARE)
Source: PLoS One. 2025 May 30;20(5):e0322089. doi: 10.1371/journal.pone.0322089 (PMC12124528; doi:10.1371/journal.pone.0322089)
Supplement: S6 Table — (DOCX) [file pone.0322089.s007.docx]

**S7 Table. Linear regression in wave 8 with covariates**

| Model | B | SE | Beta | t | p | 95.0% CI for B | |
| --- | --- | --- | --- | --- | --- | --- | --- |
|  |  |  |  |  |  | Lower | Upper |
| Constant | 40.62 | 2.36 |  | 17.24 | **<0.001** | 35.97 | 45.27 |
| Country | -0.08 | 0.03 | -0.16 | -2.87 | **0.005** | -0.13 | -0.03 |
| SRH | -1.34 | 0.45 | -0.18 | -2.96 | **0.004** | -2.23 | -0.45 |
| EURO-D | -1.07 | 0.15 | -0.45 | -7.33 | **<0.001** | -1.36 | -0.78 |
| BFI - Extraversion | 0.64 | 0.34 | 0.10 | 1.90 | 0.06 | -0.03 | 1.30 |
| BFI - Agreeableness | 0.73 | 0.38 | 0.10 | 1.92 | 0.06 | -0.02 | 1.49 |
| IADL | -0.62 | 0.22 | -0.16 | -2.78 | **0.006** | -1.06 | -0.18 |

Dependent Variable: CASP, n = 195

adjusted R^2^ = 0.46, F(6, 188) = 28.55, p < 0.001; Durbin-Watson = 1.58

Note: BFI = Big Five Inventory; CASP = Control, Autonomy, Self-realization, Pleasure (QoL) Score; CI = Confidence Interval; EURO-D = depressive symptoms questionnaire; IADL = instrumental activities of daily living; SE = Standard Error; SRH = self-rated health
