# Supplementary material for: The influence of the Big Five inventory on quality of life in people with Parkinson’s disease aged 50 and above: A Longitudinal Analysis from the Survey of Health, Aging and Retirement in Europe (SHARE)
Source: PLoS One. 2025 May 30;20(5):e0322089. doi: 10.1371/journal.pone.0322089 (PMC12124528; doi:10.1371/journal.pone.0322089)
Supplement: S7 Table — (DOCX) [file pone.0322089.s008.docx]

**S8 Table. Linear Regression with Imputed Data (Wave 7) and Covariates**

| **Imput** | **Model** | **B** | **SE** | **Beta** | **t** | **p** | **95.0% CI for B** | |
| --- | --- | --- | --- | --- | --- | --- | --- | --- |
|  |  |  |  |  |  |  | **Lower** | **Upper** |
| 1 | Constant | 38.66 | 2.13 |  | 18.13 | **<0.001** | 34.47 | 42.85 |
|  | Country | -0.08 | 0.02 | -0.15 | -4.53 | **<0.001** | -0.11 | -0.04 |
|  | SRH | -1.05 | 0.29 | -0.13 | -3.59 | **<0.001** | -1.62 | -0.47 |
|  | EURO-D | -0.82 | 0.09 | -0.34 | -9.46 | **<0.001** | -0.99 | -0.65 |
|  | BFI – Extraversion | 0.47 | 0.22 | 0.07 | 2.14 | **0.03** | 0.04 | 0.91 |
|  | BFI – Agreeableness | 0.56 | 0.25 | 0.08 | 2.26 | 0.07 | 0.07 | 1.05 |
|  | BFI – Conscientiousness | 0.48 | 0.24 | 0.07 | 2.00 | **0.04** | 0.01 | 0.96 |
|  | BFI – Neuroticism | -1.27 | 0.21 | -0.21 | -6.08 | **<0.001** | -1.68 | -0.86 |
|  | BFI – Openness | 0.65 | 0.21 | 0.10 | 3.07 | **0.002** | 0.23 | 1.07 |
|  | Mobility | -1.03 | 0.18 | -0.22 | -5.78 | **<0.001** | -1.38 | -0.68 |
|  | Recall | 0.32 | 0.11 | 0.10 | 2.78 | **0.006** | 0.09 | 0.54 |
|  | F (10, 457) = 49.75, p < 0.001, adjusted R^2^ = 0.51, Durbin-Watson = 1.67, n = 468 | | | | | | |  |
| 2 | Constant | 38.09 | 2.16 |  | 17.64 | **<0.001** | 33.85 | 42.34 |
|  | Country | -0.07 | 0.02 | -0.13 | -4.00 | **<0.001** | -0.10 | -0.04 |
|  | SRH | -0.92 | 0.30 | -0.12 | -3.07 | **0.002** | -1.51 | -0.33 |
|  | EURO-D | -0.75 | 0.09 | -0.30 | -8.26 | **<0.001** | -0.93 | -0.57 |
|  | BFI – Extraversion | 0.56 | 0.22 | 0.08 | 2.50 | **0.01** | 0.12 | 0.99 |
|  | BFI – Agreeableness | 0.52 | 0.25 | 0.07 | 2.06 | 0.05 | 0.02 | 1.02 |
|  | BFI – Conscientiousness | 0.51 | 0.25 | 0.07 | 2.07 | **0.04** | 0.03 | 0.99 |
|  | BFI – Neuroticism | -1.17 | 0.21 | -0.19 | -5.47 | **<0.001** | -1.59 | -0.75 |
|  | BFI – Openness | 0.53 | 0.22 | 0.08 | 2.45 | **0.01** | 0.11 | 0.96 |
|  | Mobility | -1.26 | 0.18 | -0.27 | -7.08 | **<0.001** | -1.61 | -0.91 |
|  | Recall | 0.25 | 0.12 | 0.08 | 2.13 | **0.03** | 0.02 | 0.48 |
|  | F (10, 460) = 46.55, p < 0.001, adjusted R^2^ = 0.49, Durbin-Watson = 1.80, n = 471 | | | | | | |  |
| 3 | Constant | 44.04 | 2.85 |  | 15.47 | **<0.001** | 38.44 | 49.63 |
|  | Country | -0.07 | 0.02 | -0.14 | -4.28 | **<0.001** | -0.10 | -0.04 |
|  | SRH | -0.96 | 0.29 | -0.12 | -3.38 | **<0.001** | -1.52 | -0.40 |
|  | EURO-D | -0.99 | 0.09 | -0.40 | -11.49 | **<0.001** | -1.16 | -0.82 |
|  | BFI – Extraversion | 0.41 | 0.21 | 0.06 | 1.93 | 0.05 | -0.01 | 0.83 |
|  | BFI – Agreeableness | 0.47 | 0.24 | 0.06 | 1.98 | 0.05 | 0.01 | 0.94 |
|  | BFI – Conscientiousness | 0.45 | 0.23 | 0.06 | 1.93 | 0.05 | -0.01 | 0.91 |
|  | BFI – Neuroticism | -1.10 | 0.20 | -0.18 | -5.43 | **<0.001** | -1.50 | -0.70 |
|  | BFI – Openness | 0.63 | 0.20 | 0.10 | 3.16 | **0.002** | 0.240 | 1.03 |
|  | Mobility | -1.04 | 0.17 | -0.22 | -5.98 | **<0.001** | -1.38 | -0.70 |
|  | Age | -0.05 | 0.03 | -0.06 | -1.91 | 0.06 | -0.10 | 0.01 |
|  | F (10, 455) = 54.48, p < 0.001, adjusted R^2^ = 0.54, Durbin-Watson = 1.81, n = 466 | | | | | | |  |
| 4 | Constant | 39.02 | 2.09 |  | 18.66 | **<0.001** | 34.91 | 43.13 |
|  | Country | -0.07 | 0.02 | -0.14 | -4.13 | **<0.001** | -0.10 | -0.04 |
|  | SRH | -0.77 | 0.29 | -0.10 | -2.66 | **0.01** | -1.34 | -0.20 |
|  | EURO-D | -0.89 | 0.09 | -0.37 | -10.40 | **<0.001** | -1.06 | -0.72 |
|  | BFI – Extraversion | 0.52 | 0.22 | 0.08 | 2.39 | **0.02** | 0.09 | 0.94 |
|  | BFI – Agreeableness | 0.45 | 0.25 | 0.06 | 1.83 | 0.07 | -0.03 | 0.93 |
|  | BFI – Conscientiousness | 0.43 | 0.24 | 0.06 | 1.79 | 0.07 | -0.04 | 0.90 |
|  | BFI – Neuroticism | -1.21 | 0.21 | -0.20 | -5.92 | **<0.001** | -1.62 | -0.81 |
|  | BFI – Openness | 0.47 | 0.21 | 0.07 | 2.24 | **0.03** | 0.06 | 0.88 |
|  | Mobility | -1.18 | 0.17 | -0.25 | -6.85 | **<0.001** | -1.51 | -0.84 |
|  | Recall | 0.24 | 0.11 | 0.08 | 2.13 | **0.03** | 0.02 | 0.46 |
|  | F (10, 456) = 52.15, p < 0.001, adjusted R^2^ = 0.52, Durbin-Watson = 1.82, n = 467 | | | | | | |  |
| 5 | Constant | 40.15 | 2.10 |  | 19.08 | **<0.001** | 36.01 | 44.28 |
|  | Country | -0.08 | 0.02 | -0.15 | -4.53 | **<0.001** | -0.11 | -0.04 |
|  | SRH | -1.01 | 0.28 | -0.13 | -3.57 | **<0.001** | -1.57 | -0.46 |
|  | EURO-D | -0.86 | 0.09 | -0.36 | -10.12 | **<0.001** | -1.03 | -0.69 |
|  | BFI – Extraversion | 0.33 | 0.22 | 0.05 | 1.50 | 0.14 | -0.10 | 0.75 |
|  | BFI – Agreeableness | 0.49 | 0.24 | 0.07 | 2.01 | 0.05 | 0.01 | 0.97 |
|  | BFI – Conscientiousness | 0.40 | 0.24 | 0.06 | 1.69 | 0.09 | -0.06 | 0.87 |
|  | BFI – Neuroticism | -1.26 | 0.21 | -0.21 | -6.17 | **<0.001** | -1.66 | -0.86 |
|  | BFI – Openness | 0.64 | 0.21 | 0.10 | 3.06 | **0.002** | 0.23 | 1.05 |
|  | Mobility | -1.11 | 0.17 | -0.24 | -6.43 | **<0.001** | -1.45 | -0.77 |
|  | Recall | 0.25 | 0.11 | 0.08 | 2.19 | **0.03** | 0.03 | 0.47 |
|  | F (10, 452) = 52.09, p < 0.001, adjusted R^2^ = 0.53, Durbin-Watson = 1.77, n = 463 | | | | | | |  |

Dependent Variable: CASP

Note: BFI = Big Five Inventory; CASP = Control, Autonomy, Self-realization, Pleasure (QoL) Score; CI = Confidence Interval; EURO-D = depressive symptoms questionnaire; Imput = Imputation, SE = Standard Error; SRH = self-rated health
