# Supplementary material for: The influence of the Big Five inventory on quality of life in people with Parkinson’s disease aged 50 and above: A Longitudinal Analysis from the Survey of Health, Aging and Retirement in Europe (SHARE)
Source: PLoS One. 2025 May 30;20(5):e0322089. doi: 10.1371/journal.pone.0322089 (PMC12124528; doi:10.1371/journal.pone.0322089)
Supplement: S8 Table — (DOCX) [file pone.0322089.s009.docx]

**S9 Table. Linear Regression with Imputed Data (Wave 8) and Covariates**

| **Imput** | **Model** | **B** | **SE** | **Beta** | **t** | **p** | **95.0% CI for B** | |
| --- | --- | --- | --- | --- | --- | --- | --- | --- |
|  |  |  |  |  |  |  | **Lower** | **Upper** |
| 1 | Constant | 40.83 | 2.36 |  | 17.33 | **<0.001** | 36.18 | 45.48 |
|  | Country | -0.08 | 0.03 | -0.15 | -2.82 | **0.005** | -0.13 | -0.02 |
|  | SRH | -1.40 | 0.45 | -0.19 | -3.12 | **0.002** | -2.28 | -0.51 |
|  | EURO-D | -1.08 | 0.15 | -0.45 | -7.39 | **<0.001** | -1.36 | -0.79 |
|  | BFI – Extraversion | 0.61 | 0.33 | 0.10 | 1.87 | 0.06 | -0.03 | 1.26 |
|  | BFI – Agreeableness | 0.71 | 0.38 | 0.10 | 1.85 | 0.07 | -0.05 | 1.46 |
|  | IADL | -0.57 | 0.22 | -0.15 | -2.56 | **0.01** | -1.00 | -0.13 |
|  | F (6, 193) = 28.81, p < 0.001, adjusted R^2^ = 0.46, Durbin-Watson = 1.61, n = 200 | | | | | | | |
| 2 | Constant | 40.22 | 2.48 |  | 16.19 | **<0.001** | 35.32 | 45.12 |
|  | Country | -0.07 | 0.03 | -0.13 | -2.40 | **0.02** | -0.12 | -0.01 |
|  | SRH | -1.31 | 0.44 | -0.18 | -2.96 | **0.003** | -2.19 | -0.44 |
|  | EURO-D | -1.05 | 0.14 | -0.44 | -7.31 | **<0.001** | -1.33 | -0.76 |
|  | BFI – Agreeableness | 0.82 | 0.38 | 0.11 | 2.18 | **0.03** | 0.08 | 1.56 |
|  | Recall | 0.34 | 0.21 | 0.10 | 1.68 | 0.10 | -0.06 | 0.75 |
|  | IADL | -0.50 | 0.23 | -0.13 | -2.14 | **0.03** | -0.96 | -0.05 |
|  | F (6, 193) = 28.99, p < 0.001, adjusted R^2^ = 0.46, Durbin-Watson = 1.60, n = 200 | | | | | | | |
| 3 | Constant | 40.17 | 2.49 |  | 16.12 | **<0.001** | 35.26 | 45.09 |
|  | Country | -0.07 | 0.03 | -0.13 | -2.42 | **0.02** | -0.12 | -0.01 |
|  | SRH | -1.32 | 0.45 | -0.18 | -2.97 | **0.003** | -2.20 | -0.44 |
|  | EURO-D | -1.03 | 0.14 | -0.44 | -7.19 | **<0.001** | -1.31 | -0.75 |
|  | BFI – Agreeableness | 0.83 | 0.38 | 0.12 | 2.19 | **0.03** | 0.08 | 1.57 |
|  | Recall | 0.34 | 0.21 | 0.10 | 1.67 | 0.10 | -0.06 | 0.75 |
|  | IADL | -0.50 | 0.24 | -0.13 | -2.13 | **0.03** | -0.97 | -0.04 |
|  | F (6, 193) = 28.57, p < 0.001, adjusted R^2^ = 0.45, Durbin-Watson = 1.60, n = 200 | | | | | | | |
| 4 | Constant | 40.74 | 2.34 |  | 17.42 | **<0.001** | 36.13 | 45.35 |
|  | Country | -0.08 | 0.03 | -0.16 | -2.97 | **0.003** | -0.13 | -0.03 |
|  | SRH | -1.38 | 0.44 | -0.19 | -3.11 | **0.002** | -2.25 | -0.50 |
|  | EURO-D | -1.08 | 0.14 | -0.46 | -7.65 | **<0.001** | -1.36 | -0.80 |
|  | BFI – Extraversion | 0.68 | 0.32 | 0.11 | 2.09 | **0.04** | 0.04 | 1.31 |
|  | BFI – Agreeableness | 0.70 | 0.38 | 0.10 | 1.86 | 0.07 | -0.04 | 1.45 |
|  | IADL | -0.58 | 0.22 | -0.15 | -2.68 | **0.01** | -1.01 | -0.15 |
|  | F (6, 193) = 29.78, p < 0.001, adjusted R^2^ = 0.47, Durbin-Watson = 1.59, n = 200 | | | | | | | |
| 5 | Constant | 40.75 | 2.33 |  | 17.47 | **<0.001** | 36.15 | 45.35 |
|  | Country | -0.08 | 0.03 | -0.16 | -3.09 | **0.002** | -0.14 | -0.03 |
|  | SRH | -1.43 | 0.44 | -0.19 | -3.26 | **0.001** | -2.30 | -0.57 |
|  | EURO-D | -1.06 | 0.14 | -0.46 | -7.72 | **<0.001** | -1.33 | -0.79 |
|  | BFI – Extraversion | 0.69 | 0.32 | 0.11 | 2.14 | **0.03** | 0.05 | 1.33 |
|  | BFI – Agreeableness | 0.75 | 0.38 | 0.11 | 1.99 | 0.05 | 0.01 | 1.50 |
|  | IADL | -0.59 | 0.22 | -0.16 | -2.72 | **0.01** | -1.02 | -0.16 |
|  | F (6, 193) = 30.04, p < 0.001, adjusted R^2^ = 0.47, Durbin-Watson = 1.58, n = 200 | | | | | | | |

Dependent Variable: CASP

Note: BFI = Big Five Inventory; CASP = Control, Autonomy, Self-realization, Pleasure (QoL) Score; CI = Confidence Interval; EURO-D = depressive symptoms questionnaire; IADL = instrumental activities of daily living, Imput = Imputation, SE = Standard Error; SRH = self-rated health
